# Myokine Response to Blood-Flow Restricted Resistance Exercise in Younger and Older Males in an Untrained and Resistance-Trained State: A Pilot Study

**DOI:** 10.1007/s42978-022-00164-2

**Published:** 2022-05-13

**Authors:** Dean M. Cordingley, Judy E. Anderson, Stephen M. Cornish

**Affiliations:** 1 Applied Health Sciences, University of Manitoba, Winnipeg, MB R3T 2N2 Canada; 2 Pan Am Clinic Foundation, 75 Poseidon Bay, Winnipeg, MB R3M 3E4 Canada; 3 Faculty of Science, University of Manitoba, Winnipeg, MB Canada; 4 Faculty of Kinesiology and Recreation Management, University of Manitoba, 110 Frank Kennedy Centre, Winnipeg, MB R3T 2N2 Canada; 5 Centre for Aging, University of Manitoba, Winnipeg, MB R3T 2N2 Canada

**Keywords:** Hypertrophy, Myokine, Exercise immunology, BFR, Aging

## Abstract

**Purpose:**

The purpose of this study was to examine the response of myokines to blood-flow restricted resistance-exercise (BFR-RE) in younger and older males before and after completing a 12-week resistance-training program.

**Methods:**

There were 8 younger (24.8 ± 3.9 yrs) and 7 older (68.3 ± 5.0 yrs) untrained male participants completed this study. Anthropometric and maximal strength (1RM) measurements were collected before and after a 12-week, supervised, progressive full-body resistance-training program. As well, an acute bout of full-body BFR-RE was performed with venipuncture blood samples collected before and immediately following the BFR-RE, followed by sampling at 3, 6, 24 and 48 h.

**Results:**

The 12-week training program stimulated a 32.2% increase in average strength and 30% increase in strength per kg of fat free mass. The response of particular myokines to the acute bout of BFR-RE was influenced training status (IL-4, untrained = 78.1 ± 133.2 pg/mL vs. trained = 59.8 ± 121.6 pg/mL, *P* = 0.019; IL-7, untrained = 3.46 ± 1.8 pg/mL vs. trained = 2.66 ± 1.3 pg/mL, *P* = 0.047) or both training and age (irisin, *P* = 0.04; leukemia inhibitory factor, *P* < 0.001). As well, changes in strength per kg of fat free mass were correlated with area under the curve for IL-4 (*r* = 0.537; *P* = 0.039), IL-6 (*r* = 0. 525; *P* = 0.044) and LIF (*r* = − 0.548; *P* = 0.035) in the untrained condition.

**Conclusion:**

This study identified that both age and training status influence the myokine response to an acute bout of BFR-RE with the release of IL-4, IL-6 and LIF in the untrained state being associated with changes in strength per kg of fat free mass.

## Introduction

Sarcopenia is the progressive loss of muscle mass, strength and physical performance during the aging process and is related to lower quality of life [57] and negative health outcomes [11, 25, 95, 102]. Resistance training, aerobic exercise and balance activities have been proposed as modalities to prevent and treat sarcopenia [62]. The development of blood-flow restricted resistance exercise (BFR-RE) has raised interest in its use in older adults [7, 36]. BFR-RE involves administering compression via an external cuff at the most proximal aspect of an exercising limb which impedes venous return and arterial flow [61]. While the limb is under vascular constriction the individual engages in resistance exercise, typically using a low-load and high-repetition protocol with minimal rest [70]. BFR-RE is reported to increase musculoskeletal strength and hypertrophy in both younger and older populations [7, 80]. The appeal of BFR-RE for use in an older population is the positive impact to improving hypertrophy and strength that will counteract age related declines, especially as these can be achieved with relatively lower loads compared to traditional resistance training. While these impacts of BFR-RE are important, the mechanisms underlying the associated skeletal muscle adaptations are likely unique although not fully understood.

One potential hypertrophic mechanism associated with a bout of acute resistance exercise is the secretion of muscle-derived cytokines, called myokines, which are a family of polypeptides and proteins released from skeletal muscle during contraction and allow for intracellular communication [76, 103]. Although some myokines are associated with muscle catabolism in the diseased state [6, 30, 51, 90], acute myokine secretion following resistance exercise can exert anabolic and growth-promoting characteristics [19, 53]. Interleukin-4 (IL-4), IL-6, IL-7, leukemia inhibitory factor (LIF) and irisin are all proposed contributors to hypertrophy [18, 19, 38, 59, 71, 81]. IL-4 is an anti-inflammatory cytokine which is upregulated following strength training suggesting a role in muscle hypertrophy [26, 27, 71, 100], possibly through myoblast recruitment to myotubes [39]. Following muscle contraction, IL-6 is consistently found to be increased and released systemically [19, 31], and is correlated with muscle hypertrophy in humans [59]. IL-7 mRNA expression is upregulated in human skeletal muscle following resistance training with some evidence that it may increase myoblast migration toward myotube formation [38], and act on satellite cells [68]. LIF can stimulate satellite cell proliferation through the STAT3 pathway and increase protein synthesis through activation of the mTOR pathway leading to skeletal muscle hypertrophy [34, 81]. Although primarily known for the “browning” of white adipose tissue [78], irisin also promotes muscle hypertrophy [72] by upregulating expression of insulin-like growth factor-1 (IGF-1), down-regulating myostatin expression [41], and increasing satellite cell activation, myoblast proliferation, and myotube formation [72]. It must be acknowledged that some controversy surrounds the existence, detectability and physiological value of irisin [2]. An upstream molecule of interest is nitric oxide (NO) which when released increases IL-6 and IL-8 mRNA expression [83], satellite cell activation [4], and along with its metabolite peroxynitrite, can mediate skeletal muscle protein synthesis [44]. The influence of age and training status on myokine secretion with acute exercise has not been fully elucidated [8, 20]. Circulating cytokines are dysregulated at rest with increased age [3], which could result in varying responses to acute resistance exercise with age. Some variations in cytokine and hormone response to acute resistance exercise based on training status have been examined [46, 50], but it is unknown if these are consistent with BFR-RE.

The objectives of this study were to evaluate the systemic response of myokines with putative anabolic actions and nitrate, to acute BFR-RE, and determine if the systemic myokine and nitrate response is modified by resistance training in younger and older adults. We hypothesized that both resistance training for 12 weeks and age (younger versus older adult males) would affect the release of putative anabolic myokines and nitrate following an acute bout of BFR-RE.

## Methods

### Participants

A total of 18 healthy, inactive males were recruited to participate; however, 3 were unable to complete all study requirements and were therefore removed from all analyses. The participants who completed all components of the study included 8 younger (24.8 ± 3.9 years-of-age) and 7 older (68.3 ± 5.0 years-of-age) individuals. Individuals were excluded from the study if they met any of the following criteria: (1) participated in > 1 structured bout of resistance exercise per week, (2) had previously been diagnosed with an inflammatory disease, (3) consumed any anti-inflammatory medication, (4) consumed any nutritional health products with an anti-inflammatory component, (5) had a cognitive disability or mental illness, (6) had a physical disability which would prevent them from participating in the structured resistance training program, or (7) had cardiovascular disease including peripheral arterial disease. Additionally, the potential for peripheral artery disease was determined by ensuring a normal ankle brachial index [17]. All participants also completed the Get Active Questionnaire to screen for other contraindications for participation [24]. Ethical approval was provided by the University of Manitoba Research Ethics Board, and all participants provided written informed consent.

### Research Design

**Fig. 1:**
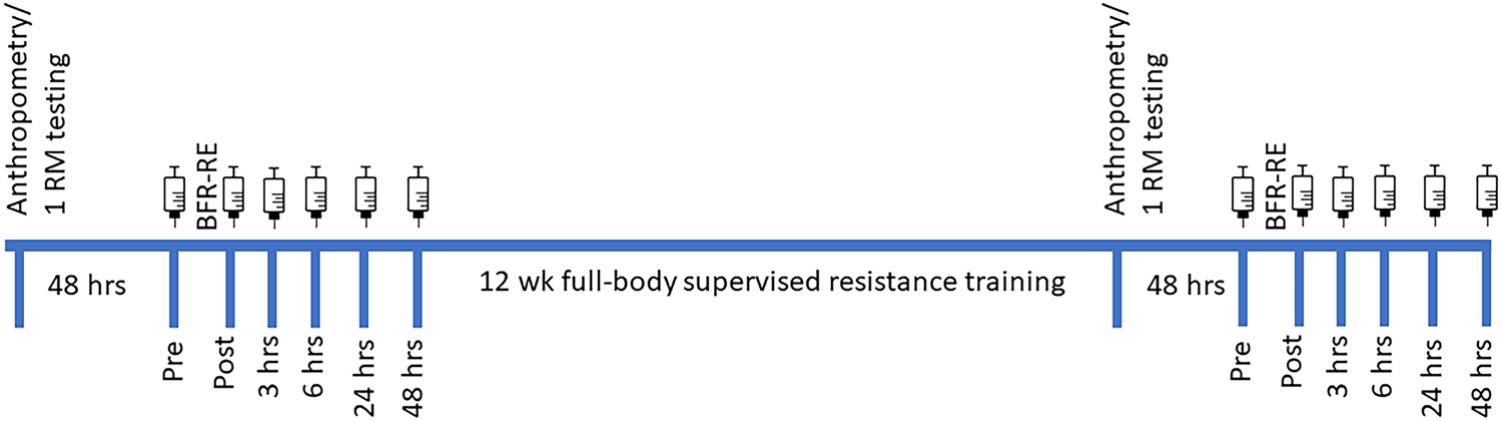
Schematic of study protocol. BFR-RE, blood-flow restricted resistance exercise. Syringe indicates blood sampling time-points

This was a non-randomized uncontrolled study. All participants reported to the research lab on 4 occasions. During visit 1, participants underwent an orientation and had their anthropometric measurements and maximal strength determined. Following a minimum of 48-h, with participants instructed not to complete any structured exercise sessions, visit 2 had participants complete an acute bout of BFR-RE while blood samples were collected pre-exercise, immediately following BFR-RE, and 3, 6, 24 and 48-h post-exercise. Following the second visit, participants underwent a 12-week full-body, supervised, resistance training program. A minimum of 48 h after the final resistance training session, visit 3 was conducted to evaluate anthropometric and maximal strength changes evoked by the training. Final assessments were made during visit 4 held a minimum or 48 h following the assessment of maximal strength, in which participants completed a second acute bout of BFR-RE and had blood sampled at the same time-points as for visit 2 (Fig. 1). During the study, participants were instructed to complete a 3-day food diary surrounding their first session of acute BFR-RE. The diaries of each participant were returned to them so they could follow the same dietary intake pattern before the final session of acute BFR-RE as a match to their own nutrient intake between the first and second visits.

### Anthropometric Measurements

Participants’ height (Seca 213, Seca, Chino, CA, USA) and body mass (BM) were determined (Seca 813, Seca, Chino, CA, USA) and body mass index (BMI) was calculated.

$$BMI=kg/{m}^{2}$$

Body fat (BF) was determined via bioelectrical impedance (InBody270, InBody Canada, Ottawa, ON, Canada) and fat mass (FM) and fat free mass (FFM) were calculated.

$$FM=kg\times BF$$

$$FFM=kg\times \left(1-BF\right)$$

### Maximal Strength Testing

All strength testing was conducted in a single exercise laboratory utilizing the same equipment. Maximal strength (1RM) was determined for chest press, leg press, seated row, leg extension and shoulder press. Prior to 1RM testing, all participants completed a 5-min cycle ergometer warm-up at a light to moderate intensity (10–13 on the 6–20 scale) as determined by the Borg Rating of Perceived Exertion. Then, the testing protocol began with participants completing 5–10 repetitions at a weight between 40% and 60% of their estimated maximum. The participants then rested for 1 min and completed 3–5 repetitions at 60%–80% of their estimated maximum followed by a 3–5 min recovery. The weight was then increased, and participants attempted 1 repetition, if successful they rested for another 3–5 min recovery period prior to increasing the weight and attempting another repetition. 1RM was determined as the heaviest weight for which the participant was able to successfully complete one full repetition of a specified exercise. Average strength (AVGSt) was calculated by summing the maximal weight lifted for all exercises and dividing by 5. Strength per kg of FFM was calculated with the following equation:

$$Strength\, per\, kg\, of\, FFM=\frac{AVGSt}{FFM}$$

### Blood Flow-Restricted Resistance Exercise Procedures

In our lab, weight plate-loaded resistance training equipment is used. Both BFR-RE sessions were conducted in the same exercise laboratory and utilizing the same equipment as the 1RM testing. A detailed description of the BFR-RE used by our lab was previously published [14]. In brief, participants were fitted with a 5-cm wide cuff (KAATSU Nano; KAATSU-Global, Huntington Beach, CA, USA) positioned at the most proximal portion of the exercising limbs (upper thighs for the lower body musculature and upper arms for the upper body musculature). The cuff was inflated until the total pressure reached 200 mmHg for the lower limb musculature [33] and 100 mmHg for the upper limb musculature [86]. The restrictive pressure of 100 mmHg or 200 mmHg (depending on the musculature being exercised) was maintained for the duration of each exercise (including rest periods), with the cuffs alternating between upper and lower limbs as appropriate. Each exercise consisted of 4 sets of 15 repetitions at 30% of the 1RM with a 30-s rest between sets and a 2-min rest between exercises. Retesting for 1RM occurred a minimum of 48 h prior to the second bout of BFR-RE to account for strength adaptations to the training protocol. Resistance exercises were always in the following order: chest press, leg press, seated row, leg extension, shoulder press and plantar flexion. Due to equipment limitations, 1RM was not determined on the plantar flexion machine; instead, the 1RM calculated from the leg press machine was used to load the plantar flexion machine to 30% of the 1RM for leg press. Although the exercises chosen were limited to those which only utilized muscle groups distal to cuff placement, they were chosen as they are common exercises performed and include the engagement of muscle groups under blood flow restriction (e.g. beyond gluteal muscles, the leg press exercise includes the activation of vastus medialis, vastus lateralis, rectus femoris, biceps femoris and gastrocnemius medialis which would all be under blood flow restriction).

### Blood Assays

All blood sampling was completed by a certified phlebotomist. Approximately 10 mL of blood was collected via venipuncture from the antecubital vein into vacutainer tubes containing ethylene diamine tetra-acetic acid (EDTA); tubes were immediately inverted multiple times to prevent coagulation. Blood was centrifuged at 1500×*g* for 15 min at 4 °C and the resultant plasma was aliquoted into microtubes and stored at − 80 °C until analysis. Myokine analysis was performed in duplicate with a Luminex MAGPIX flow cytometer (Luminex Corp., Toronto, ON, Canada). IL-4, IL-6 and IL-7 were analyzed with a Human High Sensitivity T-Cell Magnetic Bead Panel assay (Milliplex Map Kit, EMD Millipore Corp, Billerica, MA, USA). LIF and irisin were quantified with a Human Myokine Magnetic Bead Panel assay (Milliplex Map Kit, EMD Millipore Corp). Plasma nitrate/nitrite (NO_X_) was determined in duplicate using an enzyme-linked immunosorbent assay (ELISA; Nitrate/Nitrite Colorimetric Assay Kit, Cayman Chemical, Ann Arbor, MI, USA). All assays were completed according to the manufacturer’s recommendations. The intra-assay coefficient of variation for the ELISA ranged from 3.1% to 7.1% while the inter-assay coefficient was 5.93%. The coefficient of variation for the multiplex assays ranged from 7.2% to 23.8% while the inter-assay coefficient of variations ranged from 6.77% to 18.8%.

### Training Protocol

The training protocol was previously used in our lab to induce skeletal muscle adaptations in older men [21]. The training protocol was performed 3 days per week and was 12-weeks in duration. Exercises were the chest press, seated row, leg press, shoulder press, leg extension, dumbbell biceps curls, dumbbell squats, dumbbell overhead triceps press and plantar flexion. All training was conducted in the same exercise laboratory as the 1RM testing was performed. The training protocol progressed from 2 sets of each exercise, for 10 repetitions at 60% of the participants 1RM during the 1st week, to 3 sets, for 10 repetitions at 85% 1RM during the 12th week.

### Statistical Analysis

Data are presented as mean ± standard deviation (SD). Initial descriptive statistics were performed followed by a Shapiro–Wilk test to determine normal distribution; those with non-normal distribution were logarithm 10-transformed for further analysis. Variables for which logarithm 10-transformation was completed for statistical analysis are presented as absolute values to depict the physiological concentrations in circulation. An independent samples *t*-test was performed to compare the height of participants in the younger and older groups at baseline. Two-way repeated measures ANOVA (Age group × training status) with age group as a between-subject factor and training status set as the within subject factor to evaluate anthropometric, strength and strength per kg FFM changes. Three-way repeated measures ANOVA (Age group × training status × time) were conducted with age group as a between-subject factor, and training status and time set as within-subject factors to compare all biochemical variables. If Mauchly’s Test of Sphericity was violated, a Greenhouse–Geisser correction was performed. If a two-way interaction effect was detected, a Fisher’s Least Significant Difference (LSD) post hoc test was performed to determine the precise source of the significant difference. If a three-way interaction effect was detected, a break-down analysis was conducted by which the analysis output was separated by age group or training status. The resultant two-way ANOVA results were then interpreted as previously noted. Effect size is reported as partial eta squared (*η*_p_^2^) for all significant main and interaction effects. Area under the curve (AUC) for each myokine was calculated utilizing the trapezoidal method. The relationships between biochemical variables and percent change in fat-free mass and strength per kg FFM were evaluated with a Pearson correlation. All statistical analyses were performed with SPSS version 26 (IBM Corp, Armonk, N.Y., USA) with *P* < 0.05 set to indicate significance.

## Results

### Anthropometry and Muscle Strength

At baseline, there were no differences in anthropometric measures between the younger and older groups (all *P* > 0.05), although younger participants had a higher average strength (*P* = 0.019) and strength per kg FFM (*P* = 0.021). Following the 12-week resistance training intervention, there was a significant effect of training and age group on average strength and strength per kg FFM (Table 1). Additionally, fat-free mass increased (*P* = 0.031) and body fat percentage decreased (*P* = 0.030) with training (Table 1). An interaction effect was found for body mass (*P* = 0.031) and body mass index (*P* = 0.043; Table 1).

**Table 1:** Anthropometric and strength measurements before and after 12-weeks of resistance training (mean ± SD)

Variable	Younger		Older		*P*-values		
	Pre	Post	Pre	Post	Training status	Age group	Group × training status
Height (cm)	179.6 ± 11.7	N/A	177.2 ± 3.3	N/A	N/A	0.594	N/A
Body Mass (kg)	90.89 ± 20.04	92.48 ± 20.35	90.53 ± 11.28	89.29 ± 10.72	0.773	0.839	**0.031**
Body Mass Index (kg/m^2^)	28.1 ± 4.8	28.5 ± 4.5	28.8 ± 3.6	28.4 ± 3.4	0.842	0.88	**0.043**
Fat free mass (kg)	70.8 ± 12.6	72.3 ± 12.1	67.2 ± 9.1	67.9 ± 8.6	**0.031**	0.488	0.408
Fat mass (kg)	20.1 ± 11.5	20.2 ± 11.7	23.3 ± 4.4	21.4 ± 4.6	0.063	0.644	0.038
Body fat (%)	21.1 ± 8.9	20.9 ± 8.5	25.8 ± 3.8	23.9 ± 4.3	**0.030**	0.299	0.087
Average strength (kg)	103.2 ± 19.3	132.0 ± 23.7	77.0 ± 18.4	105.5 ± 27.6	**< 0.001**	**0.037**	0.937
Strength per kg FFM	1.48 ± 0.31	1.85 ± 0.35	1.14 ± 0.17	1.54 ± 0.27	**< 0.001**	**0.041**	0.647

### Blood Analysis

#### IL-4

**Fig. 2:**
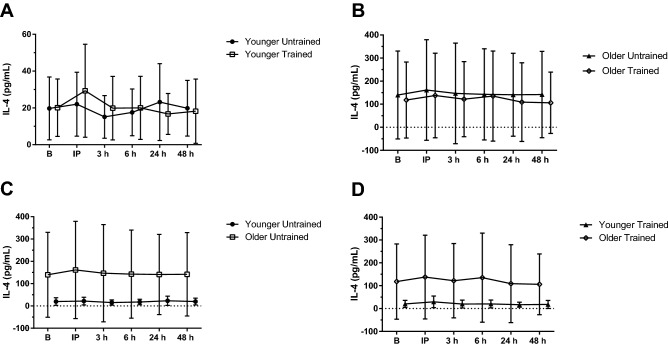
Interleukin-4 (IL-4) response to acute blood flow-restricted resistance exercise (BFR-RE) in younger (**A**) and older adults (**B**) in an untrained (**C**) and trained state (**D**; mean ± SD). *B* baseline, *IP* immediately post- BFR-RE, *3 h* 3 h following BFR-RE, *6 h* 6 h following BFR-RE, *24 h* 24 h following BFR-RE, *48 h* 48 h following BFR-RE. Significance was set at *P* < 0.05 for all comparisons

IL-4 peaked immediately following the BFR-RE to a concentration higher than all other time points (ANOVA, *P* = 0.015; *η*_p_^2^ = 0.253; *LSD*, all *P* < 0.05). An interaction effect of training status × time (*P* = 0.010; *η*_p_^2^ = 0.202) was found where IL-4 was higher 24 h post-BFR-RE in the untrained condition compared to the trained condition (*P* = 0.019). Comparisons of the age groups and training status are depicted in Fig. 2 A-D.

#### IL-6

**Fig. 3:**
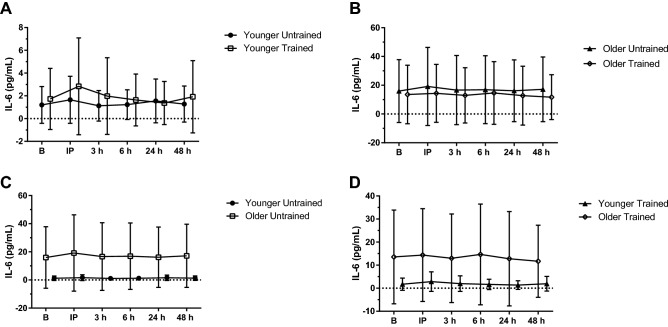
Interleukin-6 (IL-6) response to acute blood flow-restricted resistance exercise (BFR-RE) in younger (**A**) and older adults (**B**) in an untrained (**C**) and trained state (**D**; mean ± SD). *B* baseline, *IP* immediately post- BFR-RE, *3 h* 3 h following BFR-RE, *6 h* 6 h following BFR-RE, *24 h* 24 h following BFR-RE, *48 h* 48 h following BFR-RE. Significance was set at *P* < 0.05 for all comparisons

IL-6 concentrations peaked immediately after BFR-RE (*P* = 0.005; *η*_p_^2^ = 0.224) which was greater than before BFR-RE (*P* = 0.014), and 3 h (*P* = 0.004), 6 h (*P* = 0.043) and 24 h following BFR-RE (*P* = 0.003). An age group × time interaction was detected (*P* = 0.046; *η*_p_^2^ = 0.156), however pairwise comparison did not find a difference between age groups at any time-point. Comparisons of the age groups and training status are depicted in Fig. 3 A-D.

#### IL-7

**Fig. 4:**
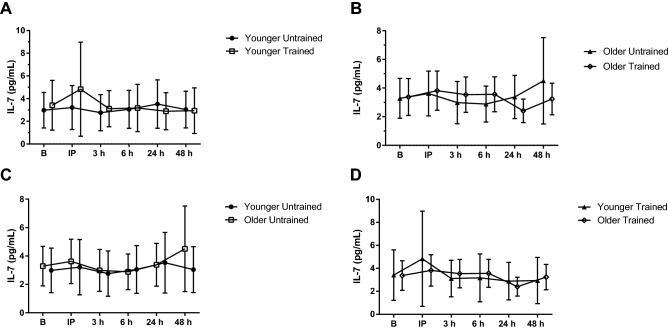
Interleukin-7 (IL-7) response to acute blood flow-restricted resistance exercise (BFR-RE) in younger (**A**) and older adults (**B**) in an untrained (**C**) and trained state (**D**; mean ± SD). *B* baseline, *IP* immediately post- BFR-RE, *3 h* 3 h following BFR-RE, *6 h* 6 h following BFR-RE, *24 h* 24 h following BFR-RE, *48 h* 48 h following BFR-RE. Significance was set at *P* < 0.05 for all comparisons

IL-7 concentrations were greater 24 h following BFR-RE in the untrained condition compared to trained (ANOVA, *P* = 0.011; *η*_p_^2^ = 0.201; *LSD*, *P* = 0.047). Comparisons of the age groups and training status are depicted in Fig. 4 A-D.

#### Irisin

**Fig. 5:**
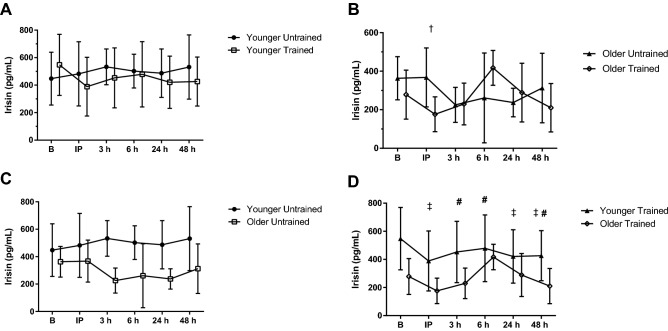
Irisin response to acute blood flow-restricted resistance exercise (BFR-RE) in younger (**A**) and older adults (**B**) in an untrained (**C**) and trained state (**D**; mean ± SD). *B* baseline, *IP* immediately post- BFR-RE, *3 h* 3 h following BFR-RE, *6 h* 6 h following BFR-RE, *24 h* 24 h following BFR-RE, *48 h* 48 h following BFR-RE. Significance was set at *P* < 0.05 for all comparisons. Dagger indicates a difference between the untrained and trained condition for the older adults. Double dagger indicates a difference between younger and older group within the trained condition. Number sign indicates different than IP within the trained condition

Younger participants had more irisin than the older group (*P* = 0.013; *η*_p_^2^ = 0.389). Irisin concentrations were greater immediately after the BFR-RE in the untrained condition compared to the trained (ANOVA, *P* = 0.018; *η*_p_^2^ = 0.186; *LSD*, *P* = 0.004). A three-way interaction was found (*P* = 0.044; *η*_p_^2^ = 0.158; Fig. 5) so breakdown analyses were conducted. When the analysis was separated by age group, older participants had greater irisin concentrations immediately following BFR-RE in the untrained condition than the trained (ANOVA, *P* = 0.024*;* *η*_p_^2^ = 0.337; *LSD*, *P* = 0.012). The analysis was then separated by training status and the younger participants had higher irisin concentrations than the older participants in the untrained condition (*P* = 0.007; *η*_p_^2^ = 0.441) and trained condition (*P* = 0.03; *η*_p_^2^ = 0.314). In the trained condition irisin at baseline was greater than concentrations immediately following BFR-RE (*P* = 0.001), and at 24 h (*P* = 0.03) and 48 h (*P* = 0.016) of recovery. Irisin 6 h after BFR-RE was greater than immediately post-BFR-RE (*P* < 0.001), 3 h (*P* = 0.031) and 48 h (*P* = 0.003) following exercise.

#### LIF

**Fig. 6:**
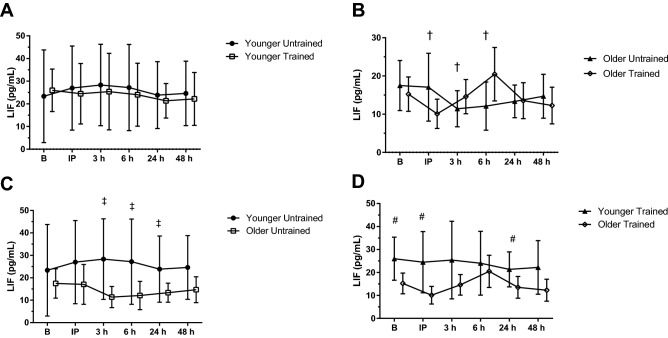
Leukemia inhibitory factor (LIF) response to acute blood flow-restricted resistance exercise (BFR-RE) in younger (**A**) and older adults (**B**) in an untrained (**C**) and trained state (**D**; mean ± SD). *B* baseline, *IP* immediately post- BFR-RE, *3 h* 3 h following BFR-RE, *6 h* 6 h following BFR-RE, *24 h* 24 h following BFR-RE, *48 h* 48 h following BFR-RE. Significance was set at *P* < 0.05 for all comparisons. Dagger indicates a difference between trained and untrained for the older group. Double dagger indicates a difference between younger and older group within the untrained condition. Number sign indicates a difference between younger and older group within the trained condition

The younger group had greater LIF than the older group (*P* = 0.038; *η*_p_^2^ = 0.292) and LIF was higher immediately following BFR-RE in the untrained condition compared to the trained (ANOVA, *P* = 0.026; *η*_p_^2^ = 0.174; *LSD*, *P* = 0.002). A three-way interaction was identified (*P* < 0.001; *η*_p_^2^ = 0.299; Fig. 6) so breakdown analyses were conducted. Pairwise comparison identified differences between older participants in the untrained and trained conditions (*P* = 0.002; *η*_p_^2^ = 0.463) immediately following BFR-RE (untrained > trained; *P* = 0.009), 3 h (untrained < trained; *P* = 0.025) and 6 h (untrained < trained; *P* = 0.029). When breakdown analysis was conducted to separate by training status, LIF was higher in the younger group compared to older in both the untrained (*P* = 0.046; *η*_p_^2^ = 0.272) and trained conditions (*P* = 0.038; *η*_p_^2^ = 0.290). In the untrained condition LIF was higher in the younger than the older group (ANOVA, *P* = 0.016; *η*_p_^2^ = 0.190) at 3 h (*P* = 0.003), 6 h (*P* = 0.017), and 24 h (*P* = 0.035) after BFR-RE in the untrained condition. In the trained condition there was an effect of time (*P* = 0.003; *η*_p_^2^ = 0.235) where LIF at baseline before BFR-RE was higher than immediately after (*P* = 0.001), at 24 h (*P* = 0.017) and 48 h (*P* = 0.019) following BFR-RE. The concentration immediately post-BFR-RE was lower than at 3 h (*P* = 0.039) and 6 h (*P* = 0.009) following BFR-RE. LIF at the 6-h time point was also higher than at 48 h following BFR-RE (*P* = 0.005). An interaction effect was detected (*P* = 0.006; *η*_p_^2^ = 0.218) whereby the younger group had greater circulating LIF prior to BFR-RE (*P* = 0.013), immediately following (*P* = 0.004), and 24 h following BFR-RE (*P* = 0.034) compared to the older group.

#### NO_x_

**Fig. 7:**
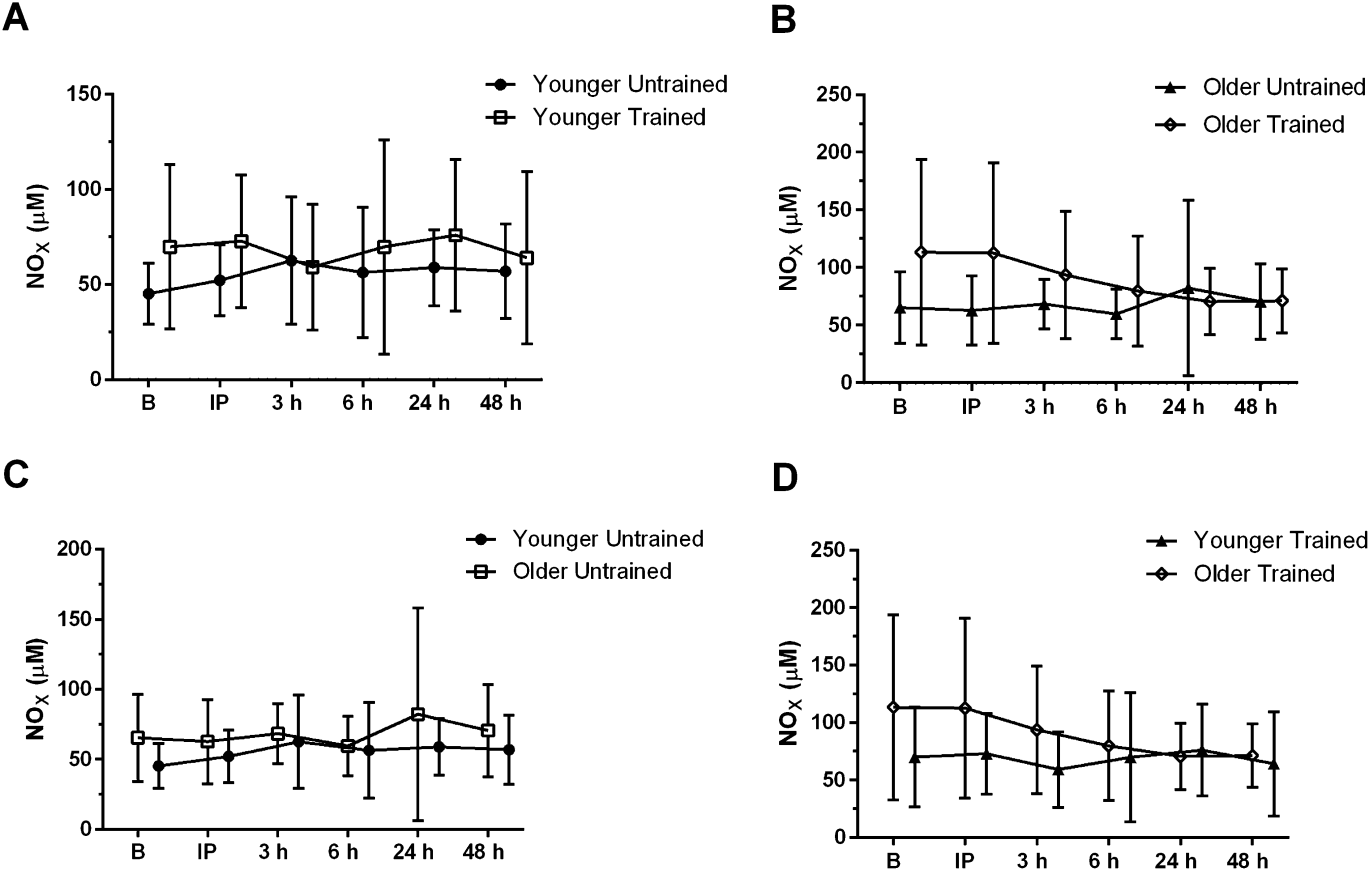
Nitrate/nitrite (NO_x_) response to acute blood flow-restricted resistance exercise (BFR-RE) in younger (**A**) and older adults (**B**) in an untrained (**C**) and trained state (**D**; mean ± SD). *B* baseline, *IP* immediately post- BFR-RE, *3 h* 3 h following BFR-RE, *6 h* 6 h following BFR-RE, *24 h* 24 h following BFR-RE, *48 h* 48 h following BFR-RE. Significance was set at *P* < 0.05 for all comparisons

There were no changes in circulating NO_X_ pre/post BFR-RE (all *P* > 0.05; Fig. 7 A-D).

### Correlations

The change in fat free mass over the 12-week training period was not correlated with any biochemical variable in the untrained or trained conditions. However, the change in strength per kg FFM was correlated with the area under the curve for IL-4 (Pearson’s *r* = 0.537; *P* = 0.039), IL-6 (Pearson’s *r* = 0.525; *P* = 0.044) and LIF (Pearson’s *r* = -0.548; *P* = 0.035) in the untrained condition.

## Discussion

The primary finding of this study was the different responses by some myokines to an acute bout of BFR-RE according to age and/or training status. The hypothesis that both training status and age would alter the release of IL-4, IL-6, IL-7, LIF, irisin and nitrate following an acute bout of BFR-RE, was only accepted for a subset of myokines. Secondary analysis also identified correlations between IL-4, IL-6 and LIF area under the curve in the untrained state and the percent change in strength per kg FFM attained from the 12-week training protocol. These results suggest potential mechanistic changes occured with age and resistance training which could contribute to variations in skeletal muscle myokine secretion in response to acute BFR-RE.

Our study identified an acute response of IL-4 following the BFR-RE, with concentrations peaking immediately following the exercise bout. These findings are supported by previous research which found IL-4 to be upregulated following a bout of resistance training [71]. As well, IL-4 concentrations were greater 24 h following the BFR-RE in the untrained condition compared to trained. Della Gatta et al. [26] also found an interaction with training for IL-4 concentrations as a 1.7-fold increase, 2 h following resistance exercise in the trained condition, possibly due to the exercise protocol using an isokinetic leg extension model, quite different from the upper and lower body BFR-RE model in the present study. As well, serial venipuncture samples were collected here, to measure circulating IL-4, while Della Gatta et al. [26] collected muscle biopsies at rest and 2 h following the isokinetic strength exercise. The systemic measures of IL-4 may not fully represent the muscular expression of this myokine locally [66].

Chronic high concentrations of circulating inflammatory signals are well-established to have negative impact on skeletal muscle [52, 58, 74, 75, 94] while acute high concentrations of inflammatory markers to exercise are beneficial [19, 67, 76]. Lavin et al. [52] showed that older adults who participate in life-long exercise are able to stave off age-related increases in circulating inflammatory signals and still maintain the acute inflammatory response to knee-extension resistance exercise [52]. IL-6 peaked immediately following the BFR-RE, indicating an acute inflammatory response to the exercise bout that did not differ between age groups or over time. Other studies investigating the response of IL-6 to an acute bout of BFR-RE also found an increase in circulating IL-6 following exercise in both young [85] and older males [65]; however, this response is not always present [14] and a decrease in IL-6 following 3 h of recovery has also been observed [63]. Therefore, variations in acute IL-6 response to BFR-RE could be due to differences in exercise volume, cuff pressure and biochemical analysis employed for quantification. With acute exercise, IL-6 is typically associated with increasing concentrations of the anti-inflammatory cytokines IL-1 receptor antagonist (ra) and IL-10 [82]. Thus, the acute response of anti-inflammatory myokines in association with exercise induced IL-6 secretion is theorized to be one mechanism whereby IL-6 exerts its communication from skeletal muscle contraction to the immune system in an endocrine manner [35]. Higher inter-individual variation in IL-6 in the older age group, consistent with findings of Della Gatta et al. [26], may have limited the ability to detect age-related differences in this particular myokine [26].

IL-7 serum concentrations have previously been found elevated immediately following a soccer match [5] indicating an acute response to an exercise stimuli. Our study did not find an acute response of circulating IL-7 following the acute BFR-RE. This difference may be due to the exercise mode, intensity, or duration. For the duration of the soccer match the participants had an average heart rate of 162 ± 2 beats/min and peak heart rate of 187 ± 2 beats/min indicating a high aerobic component to the exercise [5]. Baseline IL-7 mRNA expression has also been found to be upregulated following an 11-week strength training program [38]. In our study, there were no differences between training status conditions observed at baseline, but IL-7 concentrations were greater in the untrained condition 24 h following the acute BFR-RE. The resistance training program utilized by Haugen et al. [38] was performed 3 times per week and included 1–3 sets of leg press, leg extension, leg curl, seated chest press, seated rowing, latissimus pull down, biceps curl and shoulder press. The incorporation of all major muscle groups is similar to our program, however the lack of information regarding the intensity (percentage of 1RM), number of repetitions completed, and progression makes it difficult to compare their training program to ours.

Although irisin is primarily known for its ‘browning’ effect on white adipose tissue, recent research using meta-analysis, identified potential impacts of irisin on skeletal muscle, since acute exercise (both aerobic and resistance exercise) can increase circulating irisin [32], especially after sprint-interval exercise in young healthy males [42] and following resistance exercise [43]. It must be acknowledged that some controversy surrounds the existence, detectability and physiological value of irisin [2]. Our study found irisin levels responded to acute exercise only in the trained condition, with irisin peaking at the 6-h mark following BFR-RE, markedly delayed compared to previous studies that reported irisin peaked immediately or 30-min after exercise [42, 43]. This variation confirms a recent report that further studies should employ different types of exercise in a detailed time-course analysis [56]. Training-induced changes in irisin have not been fully elucidated [56] with one study finding increased resting irisin following 12 weeks of high-intensity interval training [47], but others not finding changes in resting irisin after 8 weeks of sprint interval training [42] or 6 weeks of whole body vibration exercise [41]. While the precise impact of exercise training on baseline irisin remains unclear, together with our study, research suggests that exercise training promotes the dynamic response and secretion of irisin following exercise.

LIF belongs to the IL-6 superfamily, as they are both structurally and functionally similar proteins [9, 13]. The expression of LIF mRNA is increased following a session of cycling at ~ 60% of VO_2max_, but this does not result in an associated increase in muscle protein concentrations [13]. Similar results have also been observed following heavy resistance exercise [12]. Broholm et al. [12] found a ninefold increase in LIF mRNA expression following resistance exercise of vastus lateralis muscle, but LIF protein was not detectable in the plasma. The results from our study detected changes in LIF with training, particularly in the older age group. The older participants responded to a bout of full body BFR-RE with concentrations of LIF immediately following the exercise bout that were higher in the untrained condition than in the trained condition. However, this relationship flipped as the participants reached 3- and 6-h following exercise where the trained condition elicited higher concentrations than the untrained. The ability of our study to detect circulating LIF compared to the aforementioned studies support the suggestion from Broholm et al. [13] that the type, and repetitiveness of the exercise stimuli are important factors to induce detectible LIF protein. Based on our findings, it appears that a blood flow restriction model with acute resistance exercise is able to stimulate a LIF response.

Nitric oxide (NO) contributes to the regulation of IL-6 and IL-8 mRNA expression in human muscle [83], and rodent models indicate NO plays a role in satellite cell proliferation [4] and the response to muscle stretch [87, 88]. Following resistance exercise, NO increases in untrained younger male adults, but only when the resistance training is performed with high loads and fewer repetitions [37]. Six months of either progressive traditional resistance training or low-load BFR-RE has been found to increase nitric oxide availability in older, hypertensive individuals with chronic kidney disease [22]. Six-weeks of aerobic exercise training can also increase circulating NO_X_ in older men and women [64]. Our study failed to demonstrate either an acute exercise or a training response in NO_X_. Variations in exercise protocols, training duration, and participant health may explain the discrepancies between studies. Rodent models investigating venous occlusion have found increased neuronal nitric oxide synthase mRNA expression that did change muscle nitric oxide content [48]. Unfortunately, this study did not include measures of gene expression and may not have detected circulating nitric oxide because of its short half-life [48, 54].

Taken together we found that in response to acute BFR-RE, concentrations of circulating irisin and LIF are greater in younger adults and decrease with age, while IL-4, IL-7, irisin and LIF concentrations decrease with 12-weeks of resistance exercise training. However, the response of IL-6 and NO_x_ did not differ between age or training conditions. The age related decrease in circulating irisin and LIF following BFR-RE could suggest they contribute to the inhibited response to resistance training, along with other physiological mechanisms such as changes in neural drive [93]. At the onset of a resistance training program there is a rapid increase in muscular strength and hypertrophy with a gradual attenuation of increases over time even when resistance training programs are maintained [73]. The attenuation of hypertrophy associated myokines (such as IL-4, IL-7, irisin and LIF) in response to resistance exercise following a period of training, may contribute to the blunted strength and hypertrophy adaptations in individuals experienced in resistance training compared to those who are resistance exercise naïve.

The change in strength per kg FFM with resistance training was positively correlated with IL-4 and IL-6 and negatively correlated with LIF. However, the older group tended to have higher IL-4 and IL-6, and lower LIF concentrations, suggesting their strength per kg FFM tended to be more favourably influenced by these myokines. Mechanistically, IL-4 appears to contribute to myoblast recruitment, formation of myotybes and is necessary for growth of muscle cells in a cell culture model [39]. IL-6 may contribute to training induced skeletal muscle adaptations in multiple manners. Evidence suggests that IL-6 secretion can induce satellite cell proliferation via the STAT3 pathway [10, 91] and could upregulate the mTOR pathway [34]. LIF is suggested to contribute to satellite cell activation [81] and increased levels of mTOR [34]. Our results support the notion that higher concentrations of IL-4 and IL-6 led to greater adaptations in strength per kg FFM, but contradict the contribution of LIF to this training induced adaptation. It could be that individuals with higher LIF in the untrained condition also had higher strength per kg FFM and therefore experienced less of a change in strength per kg FFM with training compared to those with lower LIF and lower strength per kg FFM. Recently, under restrictions related to the COVID-19 pandemic, it has been suggested that confinement in the older populations has increased sarcopenia and frailty [60, 97], this could inhibit the older populations ability to prevent infection and lead to a poor prognosis [1, 92, 96, 101]. Resistance type exercise may be of particular value for older adults to prevent pandemic restriction related muscle loss.

The technique of BFR-RE has been deemed generally safe, however the level of risk associated with this method of training is controversial [98]. It has been suggested that the potential for adverse side effects are present when implemented under certain conditions [98, 99]. Several case reports have been published indicating the occurrence of rhabdomyolysis following an acute bout of BFR-RE [16, 45, 84]. Although a relatively small number of adverse events have been reported, it is recommended that an approach which includes progressive implementation and individualized cuff pressures be implemented to limit risk [40].

Our study is not without limitations. We suggest that the response of cytokines observed in this study surrounding the BFR-RE is due to their release from exercising skeletal muscle and we therefore refer to them as myokines, however cytokines can also be secreted from other cell types which may contribute to the observed response [103]. The variability in many of the myokines was high, thus making it difficult to detect statistical differences with our sample size. Another limitation is that only systemic myokines were analyzed which does not necessarily depict the expression and protein content at the muscle level [66]. Therefore, the changes (or lack thereof) in myokine concentrations found in the blood may not depict the myokine milieu at the local level of the muscle. As well, the acute exercise response reported of the myokines in this study are specific to BFR-RE type exercise and cannot be generalized to other forms of exercise. Future research should aim to better understand the myokine response to multiple different modes, intensities and volumes of exercise in different populations, as well as include a control group participating in traditional resistance training to identify differences in myokine response specific to BFR-RE. Finally, although the study design included a request for participants to maintain the same diet in the three days leading up to the BFR-RE sessions since dietary intake can impact myokine expression [15, 77], compliance was not assessed via direct food intake analysis. As well, the cytokines measured in this study were not exhaustive and therefore other cytokines may have contributed to the observed outcomes. Specifically, the autocrine protein myostatin is of interest as it is released from skeletal muscle and negatively regulates muscle growth [28]. Myostatin is negatively associated with satellite cell activation and age-related muscle loss [79], with higher serum myostatin concentrations being associated with increased odds of sarcopenia in men [89]. Future research should evaluate the myokine response in females as they could benefit from the suggested adaptations associated with BFR-RE but are currently underrepresented in the research [23].

## Conclusion

Utilizing a blood flow-restricted resistance exercise model, this study demonstrates that training status and age can influence the acute exercise response of some myokines that are purported to have anabolic and growth-promoting characteristics. This study identified the scope of differential responses of circulating cytokines to training and age following BFR-RE. Time-dependent changes in circulating cytokines could be influenced by training and age induced changes of vascular and metabolic function. The decrease in irisin and LIF response observed with age and decrease in IL-4, IL-7, irisin and LIF with training in response to acute BFR-RE could contribute to the reported differences in musculoskeletal adaptations to resistance exercise with advancing age [29, 49, 69]and level of training [55]. These findings encourage further research on myokine responses to different exercise interventions in various human populations, including the use of nutritional interventions to modulate those myokine responses that could optimize metabolic adaptations for health and performance.

## Data Availability

Ethical approval was not obtained to provide data and material to third parties beyond that of the finalized manuscript.
